# Three Decades of China's Bt Cotton: Achievements and Insights

**DOI:** 10.1111/pbi.70641

**Published:** 2026-03-18

**Authors:** Zhigang Meng, Xiufeng Han, Guangqin Yang, Yuan Wang, Yi Zhou, Qi Zhou, Yanyan Li, Tao Zhou, Sandui Guo, Shuangxia Jin, Chengzhen Liang

**Affiliations:** ^1^ Biotechnology Research Institute (BRI) Chinese Academy of Agricultural Sciences (CAAS) Beijing China; ^2^ National Key Laboratory of Crop Genetic Improvement Huazhong Agricultural University Wuhan China; ^3^ College of Life Sciences and Technology Tarim University Xinjiang China

**Keywords:** Bt, cotton, *Helicoverpa armigera*, insect‐resistant, transgenic

## Abstract

Cotton is a vital natural fibre crop with significant economic value worldwide. In response to the threat of cotton bollworm (*Helicoverpa armigera*), the China government initiated a research project in 1992 to develop transgenic *Bacillus thuringiensis* (Bt) cotton. Through domestic research and development efforts, China achieved a significant milestone in 1994: the successful development of Bt cotton. The project has continued since then, consistently advancing agricultural plant breeding efforts and providing economically critical new cotton germplasm to combat insect infestation and other stressors. We here present an overview of the 30‐year history of this project, highlighting eight important lessons, six significant achievements, and three valuable insights gleaned from the pioneering agricultural endeavour. Chinese Bt cotton revolutionised the country's cotton industry and contributed to the economic growth and sustainability of Chinese agriculture. Through careful analysis and reflection, this article provides guidance for future development and implementation of agricultural biotechnologies.

## 1 Introduction

Cotton (*Gossypium* spp.) is a globally significant crop, serving as a primary natural fibre source for the textile industry. Cottonseed additionally provides valuable oil and animal feed (Huang et al. 2021; Wen et al. 2023). However, insect pests inflict substantial economic losses, estimated at 15%–30% (Chen, Xiao, et al. 2020; Tarazi et al. 2019). These include sucking insects (e.g., whiteflies, aphids, thrips, and jassids) and chewing insects (e.g., spiny bollworms, American bollworms, spotted bollworms, and pink bollworms), which directly damage plants and elevate production costs. Critically, many also act as vectors for plant pathogens (Rajendran et al. 2018). Over 166 insect pest species have been documented in cotton agroecosystems (Rajendran et al. 2018).

Severe infestations of cotton bollworm (*Helicoverpa armigera*) devastated Chinese cotton production during the 1990s, reducing average yields by over 40% (Cui and Guo 1996; Guo et al. 2015; Jia and Guo 2001; Zhang et al. 2007). This catastrophic decline not only caused substantial fibre shortages but also critically impacted China's textile industry, leading to reduced exports and foreign exchange revenue (Su et al. 2000). Conventional breeding approaches proved inadequate to control the escalating pest resistance (Carrière et al. 2015; Tabashnik et al. 2013; Wu et al. 2008). Faced with this challenge, Chinese scientists pioneered the development of a domestically developed insect‐resistance gene. This breakthrough enabled the successful breeding of Bt cotton varieties, which exhibited both robust yield potential and high levels of resistance against *H. armigera* (Jia and Guo 2001). These innovations represented a significant advancement in sustainable cotton production and pest management.

This review traces the development and commercialization of Bt cotton in China, extracting eight critical lessons and six major achievements. Leveraging this success, we outline three strategic research priorities for advancing cotton biotechnology globally: foundational science, intelligent design, and novel germplasm development.

## 2 Pesticidal Gene and Innovation Application

### 2.1 Pesticidal Gene Identification and Functional Analysis

**FIGURE 1:**
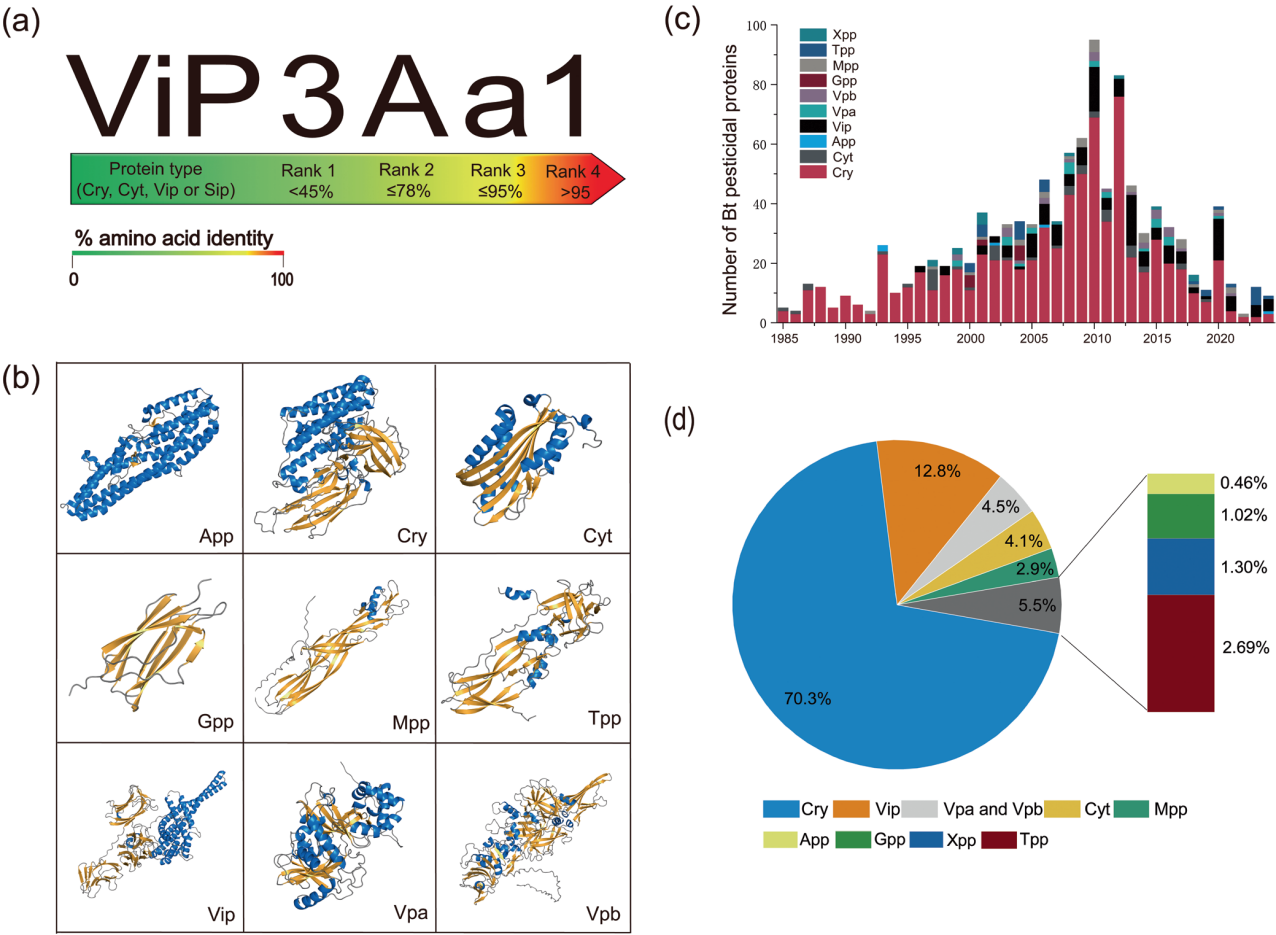
Schematic representation of Bt insecticidal proteins, nomenclature system, representative three‐dimensional structures, numbers, and proportions of different Bt insecticidal proteins classified according to the new method. (a) Schematic representation of the nomenclature system for Bt insecticidal proteins, as used by the Bt Toxin Nomenclature Committee, using Vip as an example. Each protein is given a four‐tiered name based on its amino acid sequence identity: Rank 1 involves proteins with < 45% similarity, rank 2, and rank 3 with ≤ 78% and ≤ 95% similarity, respectively, whereas rank 4 involves proteins containing > 95% homology. (b) Representative structures, where available, of the different Bt pesticidal protein classes. The data supporting these graphs come from website BPPRC (www.bpprc.org) and Unipro (https://www.uniprot.org/). (c) The number of Bt pesticidal proteins discovered and named (1985 to 2024). The data in the figure are obtained by querying BPPRC (www.bpprc.org) data and conducting statistical analysis. (d) The proportion of various proteins in Bt insecticidal protein by 2024. The data in the figure are obtained by querying BPPRC (www.bpprc.org) data and conducting statistical analysis.

The era of transgenic pest control began in 1987 with the successful introduction of a pesticidal gene into tobacco (Vaeck et al. 1987). Subsequent research has identified numerous pesticidal genes, predominantly sourced from the Gram‐positive soil bacterium Bt. Bt produces a diverse array of crystalline toxins, including crystal (Cry) and cytolytic (Cyt) proteins, as well as non‐crystalline toxins such as vegetative insecticidal proteins (Vip) and secreted insecticidal proteins (Sip). These toxins are effective against a broad spectrum of agricultural invertebrate pests, encompassing insects within the orders Lepidoptera (Syed et al. 2020), Coleoptera (Palma et al. 2014), Diptera (Soberón et al. 2018), Hemiptera (Jin, Zhang, et al. 2023; Tavares et al. 2025), Hymenoptera (Palma et al. 2014), and Orthoptera (Wu et al. 2011). These Bt toxins are recognised for their environmental compatibility and minimal impact on non‐target organisms. The canonical Cry toxins, characterised by a three‐domain structure (3D‐Cry), are traditionally classified (e.g., Cry I‐IV) on the basis of insecticidal specificity. Cyt proteins, primarily known for their toxicity against Diptera, are classified into three families: Cyt1, Cyt2, and Cyt3. Vip proteins are categorised into Vip1‐4 by sequence homology. A revised nomenclature system, defining Bt toxins by pairwise amino acid identity, was later established (Palma et al. 2014) (Figure 1a). Beyond Bt, pesticidal proteins from bacteria like *Pseudomonas aeruginosa and Bacillus cereus* (Job et al. 2022; Ulhuq and Mariano 2022) are collectively termed Bacterial Pesticidal Proteins (BPPs), targeting insects, nematodes, and mites.

Advances in genomics and structural biology prompted the Bacterial Pesticidal Protein Resource Center (BPPRC) to redefine BPPs into 16 structural types. Under this scheme, Bt pesticidal proteins are reclassified into 10 types (Crickmore et al. 2021; Schnepf et al. 1998), with structures resolved for 9 types (Figure 1b). Since 1985, the catalogue of identified Bt pesticidal proteins has steadily expanded (Figure 1c). Currently, 1139 BPPs are documented, 95.7% of which originate from Bt. Among the 1090 identified Bt pesticidal proteins, Cry and Vip types constitute 83.1% (Figure 1d). Bioassays, including membrane feeding and transgenic plant studies, confirm the variable insecticidal activities of these proteins (Table S1). For instance, Cyt1Aa inhibits *Acyrthosiphon pisum* development (Torres‐Quintero et al. 2022), Cry1F suppresses *Harmonia axyridis* populations (Paula et al. 2015), and Cry3A controls mite proliferation (Erban et al. 2009).

Cry toxins exert their insecticidal effect primarily through two distinct mechanisms: pore formation and signal transduction. In the pore‐formation model, Cry proteins bind specific receptors (e.g., cadherin, alkaline phosphatase, aminopeptidase, ATP‐binding cassette subfamily C member 2 transporter) (Liu et al. 2022; Soberón et al. 2018; Tanaka et al. 2013; Xie et al. 2005) on midgut epithelial cells, leading to pore creation, membrane integrity loss, cell lysis, and ultimately insect death (Hrithik et al. 2023; Jurat‐Fuentes et al. 2021). In contrast, the signal transduction model proposes that receptor binding initiates intracellular cascades without causing physical membrane damage (Bravo and Soberón 2008; Vachon et al. 2012). Both mechanisms initiate with proteolytic toxin activation in the insect midgut, though their relative dominance varies across species because of differences in midgut biochemistry, receptor identity, and receptor density (Dutta et al. 2022; Jin, North, et al. 2023).

Understanding these mechanisms provides a rational basis for strategic protein engineering to overcome resistance. Early work by Guo et al. created the chimeric GFM Cry1A by fusing domains from Cry1Ab and Cry1Ac (Guo 1995), a design that subsequently proved to confer both high binding affinity and intrinsic protease resistance (Guo and Cui 2000). Complementary strategies focus on disrupting key insect physiological processes required for toxin efficacy. For example, silencing midgut proteases—such as via RNAi knockdown of serine protease SfT6—can impair protoxin activation and reduce susceptibility of *Spodoptera frugiperda* to Cry1Ca1 (Rodríguez‐Cabrera et al. 2010). Similarly, impairing receptor function through CRISPR/Cas9 knockout of ABC transporters (HaABCC2, HaABCC3) in *Helicoverpa armigera* confers significant resistance to Cry1Ac (Tanaka et al. 2013; Wang, Ma, et al. 2020). Point mutations in receptors, such as T92C in HaTSPAN1, can also lead to high‐level dominant resistance (Jin et al. 2018; Li, Pang, et al. 2024).

Plant‐derived pesticidal proteins offer another avenue for resistance management. For instance, co‐expression of the cowpea trypsin inhibitor (CpTI) with Bt toxins in cotton has been used in China to create dual‐gene cultivars that delay resistance evolution in bollworms (Li, Lu, et al. 2017; Niu et al. 2020). More recently, a novel plant‐derived protein, jasmonate ZIM‐domain (JAZ) protein GhJAZ24 from cotton, was shown to bind aminopeptidase N receptors, enter cells, and induce lethality by disrupting histone deacetylase 3. Its transgenic expression confers resistance against Lepidopteran pests in multiple crops (Mo et al. 2024).

Collectively, a multipronged strategy—encompassing toxin engineering, manipulation of insect protease/receptor interactions, and deployment of plant‐derived toxins—is essential to mitigate the resistance risks exacerbated by the prolonged global use of insecticidal genes. Future efforts should therefore focus on identifying novel pesticidal genes, further rational protein design (Banerjee et al. 2022; Rausch et al. 2016; Shao et al. 2016; Walters et al. 2008), and stacking multiple effective genes in transformation vectors (Wu et al. 2021) to enhance and sustain insecticidal efficacy.

### 2.2 Innovation Application in Transgenic Bt Cotton

Because of the Bt toxin protein's ability to specifically kill target pests and its environmental friendliness, it has become the primary pesticide gene in commercialised genetically modified (GM) crops. To date, GM Bt cotton has successfully incorporated eight Bt toxin genes, including *cry1Ac*, *GFM cry1Ab/c*, *cry2Ab2*, *cry1F*, *vip3Aa*, *cry1Ab*, *cry2Ae*, and *mCry51Aa2* (https://www.isaaa.org/gmapprovaldatabase/). These toxin genes provide protection against major Lepidopteran pests, such as cotton bollworm, striped armyworm, beet armyworm, and small ground tiger, as well as Hemipteran and Thysanoptera pests, including grass blind bugs and thrips (Gassmann 2023; Guo et al. 2011; Liao et al. 2022). The successful application of these eight Bt genes has resulted in the approval of at least 4 Bt cotton events for cultivation and commercialization in major cotton‐producing countries (https://www.isaaa.org/gmapprovaldatabase/).

Chinese Bt cotton integrates a Bt gene *GFM cry1Ab/c*, which encodes a protein toxic to cotton bollworm. This variety is commonly known as Chinese Bt cotton (Guo et al. 1999, 2015). The adoption of Chinese Bt cotton has demonstrated significant effectiveness in controlling bollworm infestations, thereby reducing reliance on chemical insecticides (Huang et al. 2003; Huang, Hu, Rozelle, et al. 2002; Wu et al. 2008). These outcomes have not only enhanced cotton yields but also reduced the environmental impacts linked to pesticide use. The demonstrated success of China's Bt cotton project provides valuable insights for agricultural biotechnology (Jia and Guo 2001; Qiao 2015). These highlights emphasise the importance of robust research and development programs, collaboration among government, academia, and industry, and the necessity of intellectual property protections (Huang, Hu, and Fan 2002; Xiang et al. 2016). Over the past three decades, the Chinese government has successfully implemented several similar projects featuring innovative technological designs (Huang 2015). The remarkable success of Chinese Bt cotton has not only transformed insect‐resistant cotton breeding but also contributed to broader advancements in crop biotechnology research. Continued investment in research and development is essential to address emerging agricultural challenges, such as promoting sustainable agricultural practices, ensuring the safety of genetically modified crops, and fostering international collaboration for knowledge and technology exchange (Huang, Rozalle, et al. 2002; Pray et al. 2002; Huang et al. 2012). These efforts will contribute to enhancing global food security in a sustainable manner.

By now, the global Bt cotton cultivated area had expanded to 28 million hectares, with 12 countries exceeding 5 hectares (Tabashnik et al. 2023). The top five cotton‐producing countries—India, the United States, China, Pakistan, and Brazil—accounted for nearly 85% of the global Bt cotton planting area (Tabashnik et al. 2023). In China, millions of smallholder farmers are engaged in cotton cultivation and production (Wu et al. 2002, 2008). In 2019, India had a Bt cotton planting area of 11.9 million hectares, followed by the United States (5 million hectares), China (3.2 million hectares), Pakistan (2.5 million hectares), and Brazil (1.2 million hectares) (Tabashnik et al. 2023). Since 2019, the global planting area and yield have remained relatively stable, according to the USDA Foreign Agricultural Service (https://apps.fas.usda.gov/psdonline/app/index.html#/app/advQuery). However, the Bt cotton planting areas in the top five countries remained stable at over 95%, contributing to 76.18% of the global cotton yield in 2023. Notably, Bt cotton accounted for nearly 97% of the cotton planting area in the United States (https://www.fas.usda.gov/data/2023‐us‐agricultural‐export‐yearbook). Despite the widespread application of Bt cotton in suppressing targeted pests, long‐term efficacy is threatened in agricultural practice by the development of pest resistance (Wu and Liu 2014).

## 3 Molecular Interactions Between Cotton and Pests

Since commercial cultivation began in 1997, Bt cotton has shown excellent control effects on cotton bollworms (Wan et al. 2017). However, the rise of non‐target pests as the main pests of cotton and the enhanced resistance of cotton bollworms pose threats to the sustainable application of Bt cotton (Dhurua and Gujar 2011; Lu et al. 2020; Tabashnik et al. 2013; Xiao and Wu 2019). Therefore, we urgently need to develop a new generation of pest‐resistant solutions. Recently, the researchers have constructed the cotton genus pan‐genome and T2T‐level reference genomes (Li et al. 2021; Wang, Li, Qi, et al. 2022; Xu et al. 2025), providing crucial support for fully exploring the insect‐resistant genes in cotton germplasm resources. At the same time, the deciphering of the chromosome‐level genomes of pests such as *Apolygus lucorum* and *Adelphocoris suturalis* helped to analyse their omnivorous adaptation, drug resistance evolution, and ecological adaptation mechanisms (Liu et al. 2021; Xu et al. 2023).

### 3.1 Molecular Mechanism of Cotton Defence Responses

In recent years, the rapid development of gene editing technology has provided powerful tools for analysing gene functions, whereas the advancement of genome sequencing technology has further promoted the application of gene editing in cotton research (Li et al. 2021; Wang, Li, Qi, et al. 2022; Xu et al. 2025). Currently, researchers have developed over 10 sets of highly efficient cotton gene editing systems, which can achieve knock out, knock in, knock down, knock up, and point mutation of the target genes (Yang et al. 2025). On the basis of the bioinformatics analysis data and the transcriptome and metabolome data of cotton after being infected by pests, the researchers adopted the sgRNAs mixed library construction strategy to construct a saturated mutant library containing over 600 candidate insect‐resistant genes, and successfully obtained more than 2000 independent mutants (Sun et al. 2024; Wang, Liang, Wang, Hu, et al. 2024; Wang, Yang, Jia, Wang, et al. 2024). Through large‐scale screening, multiple key genes involved in the early defence signal transduction were identified, such as *GhGLR3.4*, *GhCPK33*, *GhCPK74*, and *GhMLP423* (Sun et al. 2024; Wang, Liang, Wang, Hu, et al. 2024; Wang, Yang, Jia, Wang, et al. 2024). The effective defence response of plants depends on the precise identification of pest damage, as well as the subsequent triggered signal transduction and cellular function reprogramming.

When plants sense insect damage, they first trigger a series of early signalling events, including depolarization of the plasma membrane transmembrane potential, increase in cytosolic Ca^2+^ concentration, reactive oxygen species (ROS) burst, and mitogen‐activated protein kinase (MAPK) cascade reaction, etc. In cotton, GhGLR3.4, as an ion channel protein, regulates Ca^2+^ influx induced by pest feeding (Wang, Yang, Jia, Wang, et al. 2024). *GhGLR3.4* also plays a significant role in facilitating the long‐distance transmission of defence signals (Wang, Yang, Jia, Wang, et al. 2024). Additionally, *GhCPK33* and *GhCPK74* jointly negatively regulate the Ca^2+^ influx induced by the oral secretions (OS) of the *Spodoptera litura* , indicating that they play a crucial role in the defence signal transduction process (Wang, Liang, Wang, Hu, et al. 2024). Another key gene, *GhMLP423*, regulates the activation of Ca^2+^ and ROS by inducing the expression of *GhEPS15* (Sun et al. 2024). Subsequently, it triggers the production of salicylic acid (SA) signals and pathogenesis‐related (PR) proteins, thereby activating the systemic defence response (Sun et al. 2024). The MAPK signalling pathway is also of great significance. Research has found that GhMPK31 interacts with the ROS‐producing protein GhRBOHB, negatively regulating the generation of H_2_O_2_ (Wang, Liang, Wang, Wang, et al. 2024). Additionally, *GhMPK3* affects the cotton's resistance to *Bemisia tabaci* by regulating the jasmonic acid (JA) and ethylene (ET) pathway mediated by MPK and WRKY transcription factors, and silencing this gene significantly enhances the cotton's susceptibility (Li et al. 2016).

Plants regulate their defence responses against herbivores through hormone signalling pathways and metabolites. The JA signalling pathway is the core pathway, and hormones such as SA and ET also play important roles. The transcriptome and metabolome analyses indicated that the OS of the *Spodoptera litura* and the *Helicoverpa armigera* could activate the cotton JA pathway and inhibit the synthesis of SA, while regulating the production of anti‐insect metabolites such as flavonoids and Gossypol (Si et al. 2020). Furthermore, the cotton plant may have specific defences against different pests: the *Helicoverpa armigera* mainly induces the JA pathway, whereas the *Apolygus lucorum* relies more on the SA pathway (Chen, Chen, et al. 2020). Plants contain tens of thousands of metabolites, some of which are toxic metabolites that have been proven to have defensive effects against pests. Gossypol is an important anti‐pest substance in cotton, having repellent and toxic effects on pests such as cotton bollworm and aphids (Gadelha et al. 2014). To reduce the toxicity of cyanogenic glycosides to humans and non‐ruminant animals, researchers used gene editing technology to knockout the key gene *GhDIR5* for the synthesis of levorotatory cyanogenic glycosides, maintaining the insecticidal ability while making cottonseed protein and cottonseed oil safer (Lin et al. 2023). The growth and development of pests such as *Spodoptera litura* , *Helicoverpa armigera* and *Apolygus lucorum* are affected by the proportion of sterol components in cotton (Lu et al. 2025). Additionally, glucosinolate (GLS) in *Arabidopsis thaliana* can also enhance resistance to cotton bollworms (Chen et al. 2025; Mao et al. 2017). Besides endogenous metabolites, the application of toxic compounds exogenously can also enhance insecticidal resistance. For instance, treating cotton leaves with caffeine can significantly enhance the toxicity against cotton bollworm larvae (Fan et al. 2025). Through the multi‐gene transformation technology, caffeine was heterologously synthesised in cotton, significantly enhancing the resistance to cotton bollworm (Fan et al. 2025). Furthermore, non‐coding RNAs also play a significant role in plant defence. For instance, miR390, lncD09, and lncA07 positively regulate the resistance of cotton to piercing‐sucking insect pests (Li, Hull, et al. 2019; Zhang, Li, et al. 2022).

### 3.2 Pests Regulation Mechanism of Cotton's Defence

In recent years, researchers have identified various effector proteins in the OS of cotton pests. These proteins interfere with plant immune signal transduction or metabolic pathways, thereby promoting pest feeding. For instance, the cotton bollworm HARP1 protein binds to plant membrane proteins CTL1, PATL2, and TET8 and then enters the cell through endocytosis, interacting with the JAZ repressor protein, inhibiting the JA signalling pathway, and thereby weakening plant defence (Chen et al. 2019; Yan et al. 2023). Similarly, the oral‐secreted effector HAS1 targets bHLH transcription factors (such as AtMYC3/4 and GoPGF), inhibiting the JA pathway and expression of defense genes, and enhancing plant susceptibility (Chen et al. 2023). The cotton bollworm effector PPI5 downregulates the expression and activity of GhFKBP17‐2, inhibiting the endoplasmic reticulum stress‐mediated immune response, and simultaneously inhibiting the JA and SA defence pathways to increase plant susceptibility (Wang, Zhu, Chen, Li, et al. 2024).

Some research progress has also been made in the oral secreted proteins of *Apolygus lucorum*. Al6 inhibits the ROS burst triggered by pathogen‐associated molecular patterns (PAMPs), weakening pattern‐triggered immunity (PTI) (Dong et al. 2020). Al106 inhibits plant E3 ubiquitin ligase PUB33 activity, thereby suppressing plant immunity (Dong et al. 2023). Interestingly, the *Apolygus lucorum* effector ASP1 exhibits a unique “ecological niche competition” strategy by binding to the transcriptional co‐repressor TOPLESS (TPL) to disrupt the inhibitory complex, activating the JA signalling and promoting gossypol synthesis, thereby inhibiting the feeding of cotton bollworms to protect its food resources (Mu et al. 2024). Plants typically recognise herbivore‐associated molecular patterns (HAMP) and damage‐associated molecular patterns (DAMP) through pattern recognition receptors (PRRs) on their cell membranes. *Arabidopsis* can perceive the OS of cotton bollworms and other Lepidoptera insects through BAK1/BIK1‐dependent PRRs (Chen et al. 2025), but the corresponding recognition mechanism in cotton remains to be elucidated.

**FIGURE 2:**
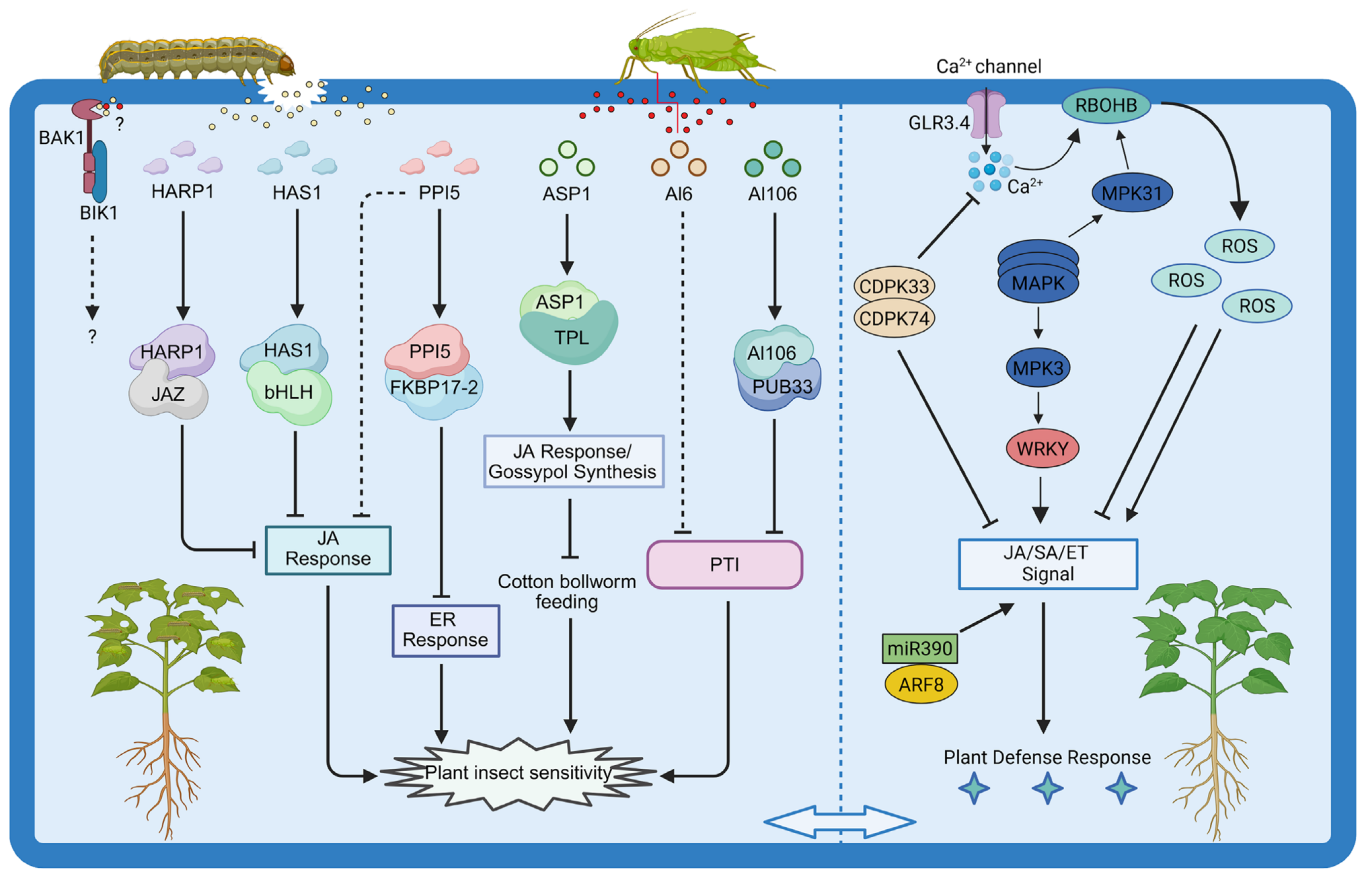
Molecular interactions between cotton and pests. Solid lines represent the proven results. Dashed lines indicate the results that have not been proven. JA, jasmonic acid. SA, salicylic acid. ET, ethylene. PTI, pattern‐triggered immunity. ER, endoplasmic reticulum. BAK1, brassinosteroid insensitive 1‐associated kinase 1. BIK1, botrytis‐induced kinase 1. JAZ, jasmonate ZIM‐domain protein. bHLH, basic helix–loop–helix. TPL, co‐repressor TOPLESS. PUB33, plant U‐box protein 33. GLR3.4, glutamate receptor 3.4. CDPK, calcium‐dependent protein kinase. MAPK, mitogen‐activated protein kinase. WRKY, WRKY transcription factor. RBOHB, respiratory burst oxidase homologue B. ROS, reactive oxygen species. miR390, miRNA 390. ARF8, auxin response factor 8. Insect oral secreted protein: HARP1, HAS1, PPI5, ASP1, Al6, and Al106.

In summary, during the long‐term evolutionary process, specific interactions have been established between pests and plants, including biochemical and behavioural adaptations, which in turn have shaped the evolution of specific plant defence regulation and expression patterns (Erb and Reymond 2019). The functional identification of anti‐pest genes and pest salivary proteins helps to better understand the complex mechanisms behind cotton defence and the adaptation mechanisms of pests to cotton (Figure 2). On the basis of these molecular mechanisms, we can use gene editing technology to mutate the pest‐susceptible genes or activate the anti‐pest genes in cotton, or silence the key genes in pests through host‐induced gene silencing (HIGS) technology to create new anti‐pest varieties and reduce cotton pest damage. In cotton, the future challenges include the discovery of new key pest‐resistant genes and further exploration of how they recognise and transmit the signals released by pests, enriching the diversity of interaction mechanisms between cotton and pests.

## 4 Eight Lessons of Chinese Bt Cotton

As described above, the achievements of China's Bt cotton program, driven by successful research and application, have spurred substantial progress in transgenic cotton research in China. However, during the research and deployment of Bt cotton varieties, eight lessons have been learned and substantial experience accumulated, informing ongoing efforts to deploy transgenic crops more effectively. In particular, the Chinese government has developed a comprehensive system comprising safety evaluation technologies for transgenic organisms and the accompanying laws and regulations. These regulatory frameworks and safety assessment technologies have enhanced public acceptance of genetically modified plants, thereby further promoting the production and application of GM crops, including GM herbicide‐resistant corn, soybeans, and cotton.

### 4.1 Demand‐Oriented Cotton Production

In the 1990s, exported textile products primarily relied on cotton fibre. However, the Chinese textile industry faced significant challenges because of a decline in cotton yield caused by the frequent and severe cotton bollworm infestations (Guo et al. 2015; Qaim and Zilberman 2003; Jia and Guo 2001). To combat this, cotton farmers resorted to excessive use of highly toxic pesticides, which increased production costs. Furthermore, indiscriminate pesticide application led to environmental degradation and contamination, posing health risks to humans and other animals throughout the food chain (Pray et al. 2001; Tudi et al. 2021). The prolonged pesticide use also led to the development of multiple pesticide resistance among cotton bollworm (Malthankar and Gujar 2016; Wu et al. 1997). Therefore, the Chinese government, cotton farmers, and textile industry professionals looked for a novel, efficient agro‐biotechnological way to overcome cotton bollworm infestations.

### 4.2 Research Objectives for Production Application

To address the significant damage caused by cotton bollworm, the Chinese government allocated funds to support GM cotton research in 1992 (Jia and Guo 2001). The primary objective of this project was to develop Chinese Bt cotton varieties with independent intellectual property rights (Huang, Rozalle, et al. 2002; Pray et al. 2002). The project deviated from traditional isolated research efforts, shifting away from hypothesis‐driven investigations and those centred on personal interests. Instead, it focused on the development of new crop germplasm with resistance to cotton bollworm that could be effectively utilised in agricultural production (Huang 2015).

Initially, China had no experience in the research and development of insect‐resistant cotton and thus had to start from scratch, resulting in a lag in Bt cotton research, development, and commercialization relative to the United States. Scepticism surrounded these efforts because of limited early successes in experimental techniques, the absence of Bt genes with independent intellectual property rights, and a shortage of molecular biology equipment at the project's outset (Huang and Zhang 2016; Shao and Chu 2005; Wang et al. 2018). Despite these challenges, the scientists maintained unwavering confidence in their ability to innovate independently and develop domestically‐produced Bt cotton varieties with independent intellectual property rights (Huang 2015; Jia and Guo 2001). This ambitious, large‐scale project thrived because its objectives were built on explicit milestones, quality metrics, and assessments. The continuous open‐mindedness and selflessness of the scientific leaders were key factors in the success of government support. Additional critical factors contributing to the project's success included the systematic evaluation of the technological roadmap and the researchers' willingness to adapt their strategies as needed.

### 4.3 Multi‐Dimensional Improvement and Innovation

**FIGURE 3:**
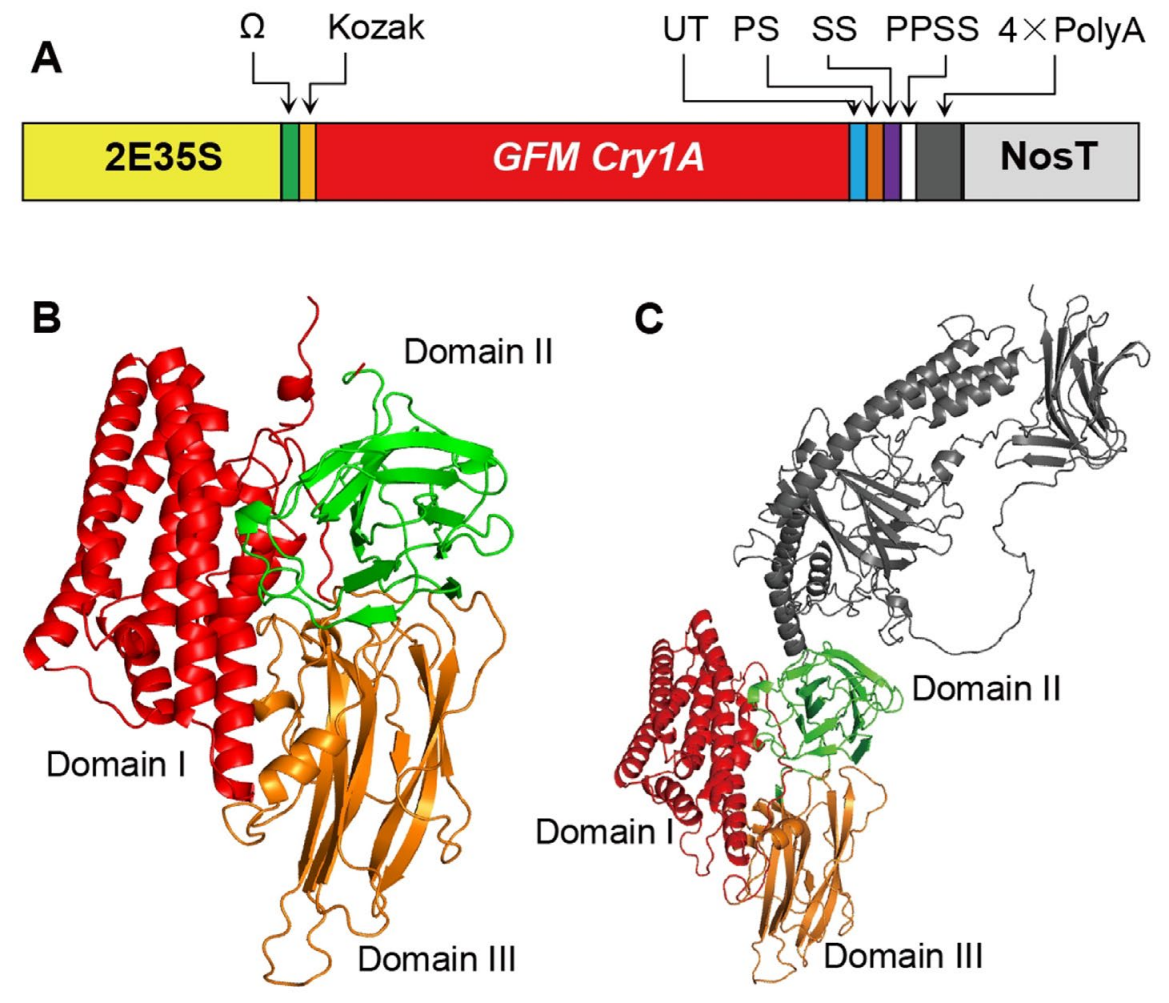
Sequence and structure of the *Bacillus thuringiensis* (Bt) protein used in Chinese insect‐resistant cotton. (a) Diagram showing the vector used for the generation of Chinese single‐gene insect‐resistant cotton. “2E35S” refers to the CaM35S promoter modified by doubling the enhancer sequence between −393 bp and −90 bp. Ω and Kozak refer to the Ω and Kozak sequences, respectively. UT, multiple stop codons. PS, processing sequence. SS, splicing sequence. PPSS, potential polyadenylation signal sequence. (b) Three‐dimensional structure of the Chinese Bt protein. (c) Three‐dimensional structure of the Bt protein engineered in the USA.

The successful development of Chinese Bt cotton can be attributed to six key technical innovations. First, a novel highly insecticidal Bt protein, GMF Cry1Ab/c, was engineered for this purpose. It combined the N‐terminal region of Cry1Ab (residues 1–286; structural domain I), which has a robust capacity to penetrate gut cells, with the C‐terminal region of Cry1Ac (residues 287–608; structural domains II and III), which has a strong binding affinity with insect gut cell membranes (Guo 1995; Endo 2022; Huang 2015) (Figure 3). The second innovation was related to specific potential polyadenylation signal sequences (PPSS) in the gene encoding Bt. These sequences can also function as polyadenylation signals in plants (Mathew et al. 2011; Misztal et al. 2004; Murray et al. 1991), leading to mRNA cleavage, which compromises gene integrity and significantly reduces gene expression levels. To address this, *GMF Cry1Ab/c* was designed with the PPSS and similar sequences removed. GFM Cry1Ab/c is thus substantially different in size compared to the insect‐resistant Cry1Ac found in American cotton because of the selective preservation of functional domains and elimination of non‐functional sequences (Figure 3b,c). Third, *GMF Cry1Ab/c* was codon‐optimised for plants to enhance its expression. To facilitate sequence modifications and codon optimisation, a comprehensive gene synthesis strategy was implemented. To ensure precise DNA synthesis, a total of 82 oligonucleotide fragments, each with a length of < 60 bp, were synthesised. These fragments were then strategically combined into nine larger fragments, incorporating artificial restriction enzyme sites at the ends (Huang 2015). Recombination techniques yielded the complete *GMF Cry1Ab/c* gene. Fourth, several modifications were made to the plant expression vector to improve *GMF Cry1Ab/c* expression, stability, and translation efficiency. These modifications included amplifying the enhancer sequence between −393 bp and −90 bp in the CaMV35S promoter to increase transcription (Odell et al. 1988); introducing a Kozak sequence (Kozak 1984) before the translation initiation codon to enhance gene expression; and incorporating a Ω sequence (Richards et al. 1978) to improve translation efficiency. Multiple stop codons were inserted at the 3′ end to prevent read‐through. Furthermore, a 4 × PolyA sequence was added before the transcription termination signal at the 3′ end to increase mRNA stability. Two splicing sequences (CATTG) (Gallie et al. 1989), one processing sequence (TGTGTTTCT) (Ingelbrecht et al. 1989), and one PPSS sequence (AAATAAA) (Ingelbrecht et al. 1989) were also included to enhance mRNA stability, prolonging the half‐life (Figure 3a). Fifth, two different genetic transformation methods, *Agrobacterium*‐mediated transformation and pollen‐tube pathway transformation (Ali et al. 2015; Ni et al. 2000), were simultaneously employed to produce Bt cotton. Last, to address a reduction in cotton bollworm resistance among Chinese Bt cotton, a line of Bt cotton that co‐expressed *GMF Cry1Ab/c* and cowpea trypsin inhibitor (CpTI) (Guo et al. 1999; Kang et al. 2005), referred to as Chinese double‐gene Bt cotton, was developed. A three‐line breeding system was also created to simultaneously increase fibre yield and insect resistance (Guo et al. 2015). These ongoing research and innovation efforts have ensured the effective performance and durability of Chinese Bt cotton varieties.

### 4.4 Collaborative Research and Development

The success of Chinese Bt cotton can be attributed to strong collaboration between scientists, government agencies, and industry stakeholders. The Chinese Bt cotton project brought together over 100 researchers from various professional cotton research institutes and breeding companies. They were divided into four subgroups, each with a specific goal. The first subgroup focused on cloning insect‐resistance genes from Bt and developing a plant vector with high expression efficiency for the gene encoding Bt. The second subgroup was responsible for genetically transforming cotton plants with those Bt genes to create Bt cotton germplasm. The third subgroup primarily focused on breeding new Bt cotton varieties using the Chinese Bt cotton germplasm created by the second subgroup. These three subgroups were populated primarily by scientists from research institutes. Members of the fourth subgroup were from the crop seed industry and were responsible for promoting and applying the Chinese Bt cotton varieties in the field. To ensure the insect resistance of the cotton lines developed by these subgroups, an independent assessment team was included in the project. Collaboration between researchers from different subgroups was common.

Especially, early Chinese Bt cotton varieties faced issues such as premature senescence, reduced Bt protein expression during later growth stages, and low yields, which significantly limited commercial deployment. However, through the collaborative efforts of the institutions cited above, these problems have largely been resolved to date (Zhang and Dong 2024).

### 4.5 Knowledge Sharing and Capacity Building

The research group of government funding revolutionised the standard practices of scientific research sharing within the field of agricultural technology research. Each of the four subgroups had a specific responsibility, ensuring a streamlined workflow (Center et al. 2016). The first subgroup identified, artificially designed, and synthesised novel insect‐resistant genes and constructed highly efficient expression vectors. The remaining groups actively engaged in collaborative initiatives with the first subgroup, offering crucial technical support and providing valuable feedback. This collaborative effort ensured a well‐rounded, scientifically rigorous approach to the project. Importantly, the subgroups actively shared research data and cotton resources with each other, facilitating a constant exchange of information that accelerated the overall project progress. This collaborative approach ensured that advances made by one subgroup could be quickly evaluated and integrated by the others. In addition to scientific achievements, the research group recognised the significance of knowledge sharing and capacity building for farmers. By disseminating scientific knowledge and empowering farmers with skills and information, the project aimed to facilitate the successful adoption and sustainable cultivation of Chinese Bt cotton. This not only benefited the farmers but also contributed to the overall development of China's agricultural sector. The collaborative framework with four subgroups, each focusing on a specific aspect of research and development, and the active exchange of research data and resources between the subgroups greatly contributed to the progress of this project. Furthermore, the emphasis on knowledge sharing and capacity building was crucial to ensuring successful, sustainable Bt cotton cultivation in China.

### 4.6 Environmental Monitoring and Risk Assessment

Monitoring and assessment of the environmental impacts of Chinese Bt cotton cultivation have played pivotal roles in ensuring long‐term sustainability. During the early stages of Bt cotton research, development, and commercial application in China, there was no established framework of technologies or regulations for the safety evaluation and supervision of genetically modified crops. It was through the deployment of Bt cotton that a relatively comprehensive system, encompassing safety evaluation technologies and accompanying laws and regulations, was gradually developed and later extended to other GM crops. Significant research has been conducted to evaluate the effects of Chinese Bt cotton on non‐target organisms, soil health, and biodiversity (Jia and Guo 2001; Wang and Li 2000; Wu et al. 2023; Xu et al. 2012). The studies demonstrated that Chinese Bt cotton cultivation has minimal detrimental effects on the environment. Targeted expression of the Chinese insect‐resistant toxin protein in cotton plants effectively controls cotton bollworm, reducing reliance on chemical insecticides and minimizing the negative impacts of such compounds on beneficial insects and other non‐target organisms (Lu et al. 2012; Wu et al. 2008; Wu and Liu 2014; Zhang et al. 2018). Moreover, Chinese Bt cotton positively influences soil health by promoting increased microbial activity and levels of organic matter (Li et al. 2011). Furthermore, comprehensive studies have indicated that Chinese Bt cotton cultivation does not significantly impact biodiversity; no adverse effects have been observed in non‐target insects, birds, or mammals (Li et al. 2011; Lu et al. 2012; Wang, Yan, et al. 2021; Zhang et al. 2018). Overall, the rigorous monitoring and risk assessment performed in Chinese Bt cotton cultivation provided valuable insights into its environmental impacts and greatly contributed to its sustainable integration into agricultural practices.

### 4.7 Long‐Term Planning and Investment

Long‐term planning and sustained investment have been crucial to the success of Bt cotton in China. In addition to the unwavering financial support from the government, various projects have provided funding for the research and industrialization of Bt cotton (Guo et al. 2015; Huang 2015). For example, Three China Ministries and One Commission have funded the development and commercialization of Chinese single‐gene Bt cotton. Chinese dual‐gene Bt cotton development and commercialization have received support from the China Agricultural Science and Technology Education Fund, and the research and commercialization of three‐line Bt cotton have been funded by the National Natural Science Foundation (Huang, Rozalle, et al. 2002; Pray et al. 2002; Huang et al. 2012). The government has also facilitated the establishment of state‐of‐the‐art molecular biology laboratories and the acquisition of advanced laboratory equipment. To meet the diverse research and development requirements of different cotton‐growing regions, multiple demonstration and trial bases for Bt cotton have been established nationwide (Center et al. 2016; Huang 2015). Moreover, substantial investments have been made in the development of new Bt cotton varieties and rigorous evaluation of their efficacy. The government has implemented comprehensive policies and regulations to ensure safe, responsible adoption of Bt cotton (Center et al. 2016). This long‐term planning and sustained investment have enabled the successful development and widespread adoption of Bt cotton in China.

### 4.8 Adaptive Management

Flexible, efficient, and adaptive management strategies, particularly for leveraging financial and property rights, allowed challenges and issues in the development of Bt cotton to be addressed in a timely fashion (Center et al. 2016). Utilising financial leverage, decision‐makers and chief scientists adjusted resource allocation and investment priorities on the basis of the specific needs of the project, ensuring optimal resource utilisation and efficient project advancement. The utilization of property rights leverage ensured the establishment of clear ownership and usage rights for each technological advance, facilitating effective coordination and cooperation among stakeholders and safeguarding the interests of researchers and project participants. Additionally, the implementation of robust feedback mechanisms and monitoring systems enabled detection of changes in the cotton industry and market in addition to timely establishment of relevant responses. Aligning Chinese Bt cotton cultivation with market demands and consumer preferences facilitated its adoption and commercial success. Flexibility and adaptability in management strategies have proven to be essential in navigating uncertainties during the course of the project, maximizing successful implementation and industrialization of Chinese Bt cotton.

## 5 Six Achievements of Chinese Bt Cotton

Over more than three decades of Bt cotton development in China, success has been achieved after overcoming numerous setbacks. In particular, early Bt cotton varieties faced several production challenges, including reduced Bt protein expression during the reproductive stage, premature senescence resulting from potassium deficiency, and bollworm resistance to Bt cotton (Zhang and Dong 2024). Through the collaborative efforts of scientists, breeders, and policymakers, these challenges have been progressively addressed, and there are six experiences gained that provide guidance for the development and deployment of new GM crops.

### 5.1 Solving the Cotton Bollworm Problem

**FIGURE 4:**
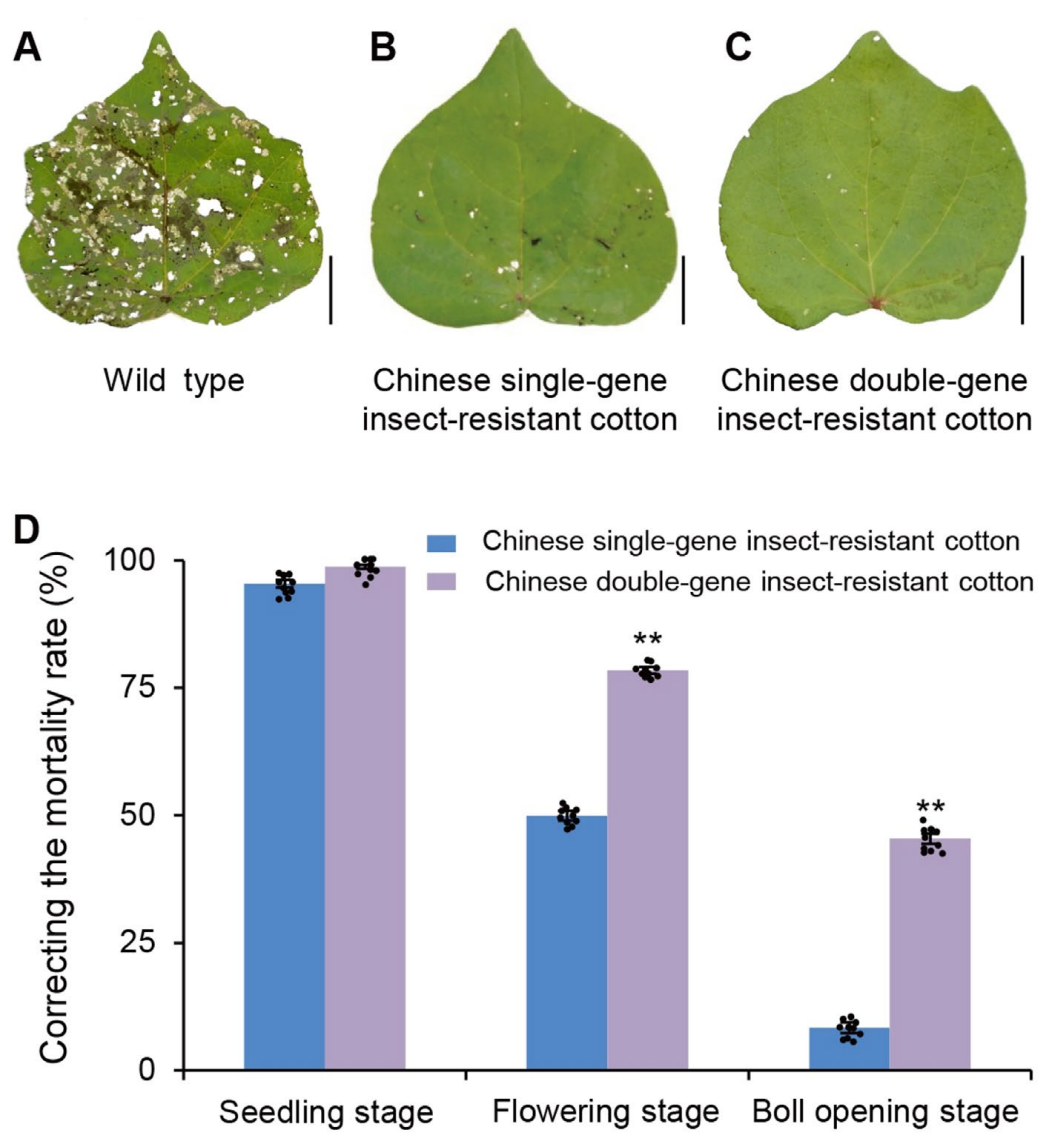
Efficacy of cotton bollworm resistance. (a–c) Representative leaves from (a) wild‐type, (b) single‐gene insect‐resistant, and (c) double‐gene insect‐resistant cotton exposed to cotton bollworm. Scale bar = 2.0 cm. (d) Corrected mortality rates of cotton bollworm exposed to Chinese single‐gene and double‐gene insect‐resistant cotton at various plant developmental stages. Bars represent the mean values from 10 independent biological replicates. **p < 0.01 (two‐tailed two‐sample *t*‐test).

The cultivation of Chinese Bt cotton has successfully solved the problem of cotton bollworm infestations. After consumption by cotton bollworm larvae, Bt protein binds to receptors on the microvilli of their intestinal epithelial cells. This interaction triggers a series of reactions, resulting in the formation of permeable channels on midgut cell membranes. These channels cause damage and shedding of the midgut cells, and larvae ultimately cease feeding and die (Bravo et al. 2011; Pardo‐López et al. 2013; Syed et al. 2020). Chinese single‐gene Bt cotton, which contains *GFM Cry1A*, exhibits > 80% insect resistance (Figure 4) and possesses complete independent intellectual property rights (Guo 1995). In 1997, five domestically developed Chinese single‐gene Bt cotton varieties (GK1, GK12, GK19, GK22, and Jinmian26) were extensively promoted, resulting in their broad adoption in provinces including Anhui, Shandong, Hubei, Jiangsu, Shanxi, Henan, and Hunan (Guo et al. 2015; Jia and Guo 2001; Sun et al. 2016; Zhang et al. 2007). This achievement positioned China as the second country (following the United States) to possess independent intellectual property rights for Bt cotton. Chinese double‐gene Bt cotton, which supplements *GFM Cry1A* with *CPTI*, has > 90% insect resistance (Guo et al. 2015; Zhang et al. 2007). Moreover, compared to Chinese single‐gene Bt cotton, it significantly increases the corrected mortality rate of cotton bollworm in the later stages of plant growth (by over 35%) and reduces the average number of cotton bollworm larvae per hundred plants in cotton fields by 57.1% (Figure 4). This effectively addresses the reduced insect resistance observed in the later stages of Chinese single‐gene Bt cotton growth (Xia and Guo 2001). In addition, Chinese double‐gene Bt cotton effectively slows the development of resistance among cotton bollworms. Chinese double‐gene Bt cotton thus established China as a global leader in Bt cotton research (Li, Lu, et al. 2017; Wang and Guo 1999).

Because of the open sharing of domestic Bt cotton germplasm resources, resistant cotton was rapidly adopted for industrial applications. Currently, there are over 500 certificates for the safe application of domestic Bt cotton; more than 200 new varieties of Bt cotton have been developed; and the cumulative planting area has reached 34 million hectares (Guo et al. 2015). Since the widespread adoption of domestically‐developed Bt cotton, China has not experienced any significant outbreaks of cotton bollworm (Wang, Xu, Huang, et al. 2022; Wang, Xu, et al. 2020; Jin et al. 2015). Thus, the successful implementation of domestically‐produced Bt cotton has effectively mitigated the threat posed by cotton bollworm in China (Qiao et al. 2017).

Moreover, with the extensive planting of Bt cotton in China, bollworms face a potential risk of evolving resistance to Bt cotton. However, resistant bollworms have not yet been observed in fields across the country. A primary factor contributing to this is the proximity of Bt cotton fields to non‐Bt crops such as corn, peanuts, soybeans, and vegetables, which creates a landscape‐level natural refuge that sustains populations of susceptible bollworms and thereby delays the emergence of Bt resistance in bollworm populations in the field (Zhang and Dong 2024).

### 5.2 Reduced Pesticide Use

The widespread utilisation of Chinese Bt cotton has resulted in a significant reduction in pesticide usage. The cultivation of Chinese Bt cotton decreases chemical pesticide usage by over 50% compared to conventional cotton fields, resulting in a cumulative reduction in pesticide usage of over 650 000 tons to date (Guo et al. 2015; Huang, Hu, Rozelle, et al. 2002; Sun et al. 2016). This substantial decrease in chemical pesticide usage in cotton fields has led to several notable benefits to the ecological environment and to human and animal health. First, the decrease in chemical pesticide usage has significantly increased the biodiversity index of cotton field ecosystems, enhancing ecological stability (Qaim 2020; Qiao et al. 2016). Populations of broad‐spectrum natural enemies, such as ladybugs, lacewings, and spiders, have substantially increased (Cui et al. 2004; Lu et al. 2012). This has greatly improved the natural pest control capacity of not only cotton fields, but also of neighbouring crops such as corn and soybean. Second, the widespread cultivation of Chinese Bt cotton has resulted in a significant reduction in the cotton bollworm population (Sun et al. 2016; Wu et al. 2008). Consequently, there has been an extended decline in pesticide use for cotton bollworm control over time, and both Bt cotton and conventional cotton have demonstrated a decreased reliance on chemical insecticides for cotton bollworm control (Hossain et al. 2004). Third, the significant reduction in pesticide application to Bt cotton fields has effectively decreased pesticide residues in the soil. This reduction has played a crucial role in mitigating environmental pollution of groundwater and river ecosystems; furthermore, it has reduced the number of poisoning incidents among animals present in the environment (Kouser and Qaim 2011; Hossain et al. 2004). Thus, the use of Bt cotton has contributed to environmental safeguarding. Last, the decrease in pesticide usage has not only increased the economic benefits of cotton to farmers, but has also reduced farmers' direct exposure to chemical pesticides, promoting their physical health (Kouser and Qaim 2011; Hossain et al. 2004). Indeed, studies have indicated that the proportion of pesticide poisoning cases caused by pesticide application has been reduced by ~10% among farmers cultivating Bt cotton compared to those cultivating conventional cotton (Huang et al. 2003). Since the widespread application of Chinese Bt cotton, there have been no large‐scale incidents of pesticide poisoning in humans or livestock in cotton fields (Hossain et al. 2004).

### 5.3 Increased Cotton Yield

Chinese Bt cotton exhibits significantly increased yield compared to conventional cotton, ensuring food security and economic prosperity. Field cultivation of Bt cotton significantly reduces the damage inflicted by cotton bollworm on cotton nutritional and reproductive organs, such as the tender leaves, flower buds, flowers, and green bolls (Sun et al. 2016; Syed et al. 2020; Xu et al. 2012). It also decreases bud shedding and the occurrence of boll rot, maximising cotton yield potential. During the initial phase of Chinese Bt cotton adoption throughout the country, Bt cotton demonstrated a consistent, significant yield increase of 8%–15% compared to conventional cotton (Fan 2005; Sun et al. 2016). In addition, the three‐line Bt cotton technology further increased both insect resistance and yield (Wang et al. 2005). Yinmian2 (Zhang et al. 2012), a three‐line GM cotton accession, was successfully developed in 2005. In field trials, it demonstrated a yield increase of > 25% compared to the control variety, making it the first internationally commercially applied three‐line transgenic Bt cotton variety. Such lines show ~20% higher seed production, ~50% higher seed efficiency, and ~60% lower production costs than conventional cotton, with 98–100% seed purity (Sun et al. 2016; Zhang et al. 2012).

**FIGURE 5:**
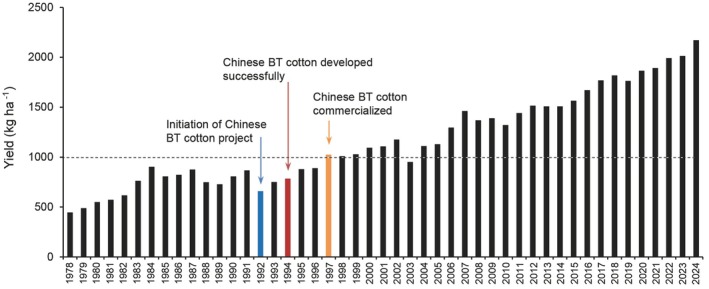
Cotton yield in China over time. Key points in Chinese insect‐resistant cotton development are noted. Data were obtained from the National Bureau of Statistics of China (http://www.stats.gov.cn/).

**FIGURE 6:**
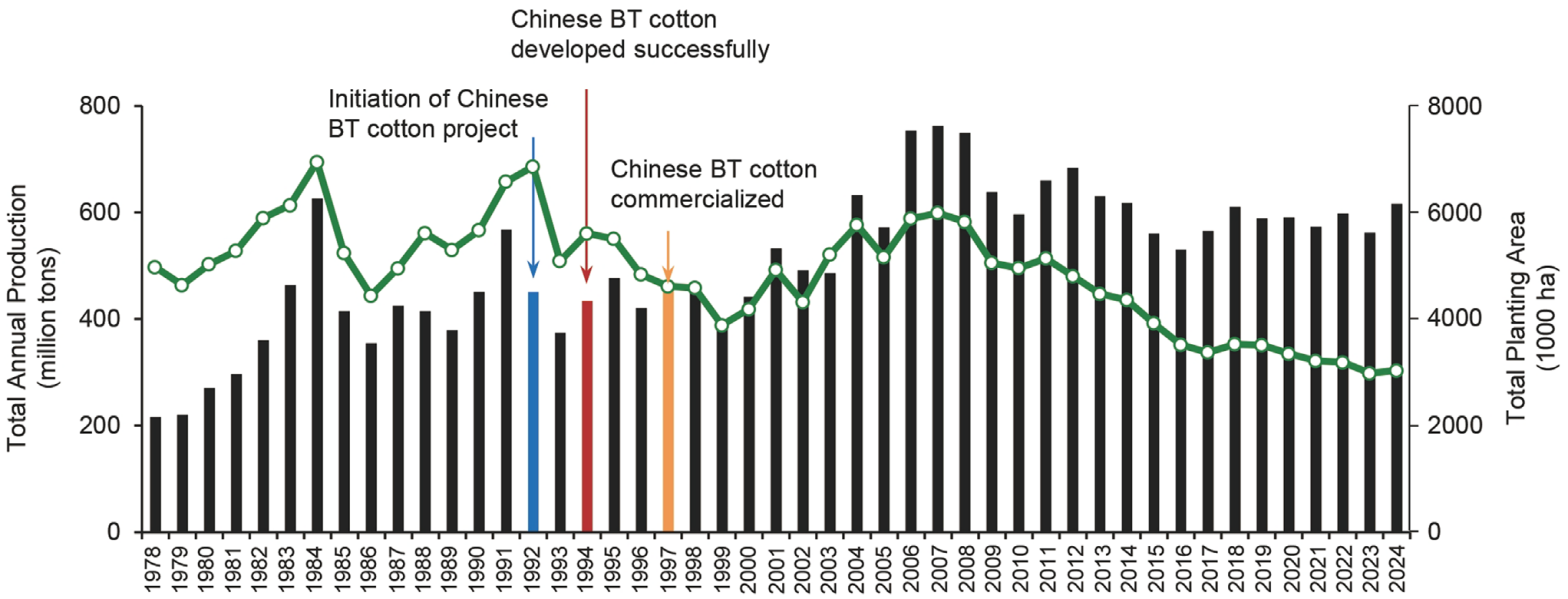
Total cotton production and total cotton planting area in China over time. Bars represent the total cotton planting area; points represent total cotton production. Data were obtained from the National Bureau of Statistics of China (http://www.stats.gov.cn/).

Following the commercialization of Chinese Bt cotton, China's cotton yield per hectare surpassed 1000 kg in 1997 (Figure 5). Through the continuous efforts of cotton researchers in China and the expanding cultivation of Bt cotton, yield per hectare has continued to increase, reaching ~1990 kg per hectare in 2022 (https://www.stats.gov.cn/sj/zxfb/). Despite a decrease of ~1 500 000 ha in the cotton planting area over the past decade, total annual cotton production has remained higher than 5 million tons, with no significant declines (Figure 6). This can be partially attributed to the extensive cultivation of domestically produced Bt cotton, which has reduced the demand for land for cotton cultivation (Qiao et al. 2016). Thus, the use of Chinese Bt cotton effectively conserves agricultural land in China, expanding the food crop planting area and ensuring domestic food security.

### 5.4 Enhanced Farmer Livelihoods

The cultivation of Chinese Bt cotton has significantly improved economic conditions for farmers, increasing their incomes and improving their livelihoods. Planting Chinese Bt cotton not only reduces pesticide application, which decreases cotton production costs and increases yield, but also leads to a substantial decrease in labor costs (Huang, Rozalle, et al. 2002). Conventional cotton cultivation requires six times more labor compared to Bt cotton cultivation; this is due to the need for pesticide application, which results in a difference of 120 d of labor per hectare (Fan 2005; Su et al. 2000). Bt cotton also requires planting of ~50%–83% fewer seeds than conventional cotton, which also contributes to decreased production costs (Fan 2005; Su et al. 2000). In addition, relief from the laborious and time‐consuming task of controlling cotton bollworms allows farmers to allocate more of their time to profitable activities that generate additional income. To date, the promotion of Chinese Bt cotton cultivation has driven a cumulative increase in agricultural output that has a value exceeding 100 billion RMB (Guo et al. 2015; Sun et al. 2016). Furthermore, the reduced frequency of pesticide application significantly reduces the direct exposure of cotton farmers to pesticides, resulting in a notable decrease in pesticide poisoning incidents (Kouser and Qaim 2011). Moreover, decreased pesticide application is associated with significant reductions in the volume of pesticide residue entering the atmosphere, soil, water, and other regions of the environment, effectively enhancing overall environmental health (Huang et al. 2003).

### 5.5 Global Influence and Cooperation

**FIGURE 7:**
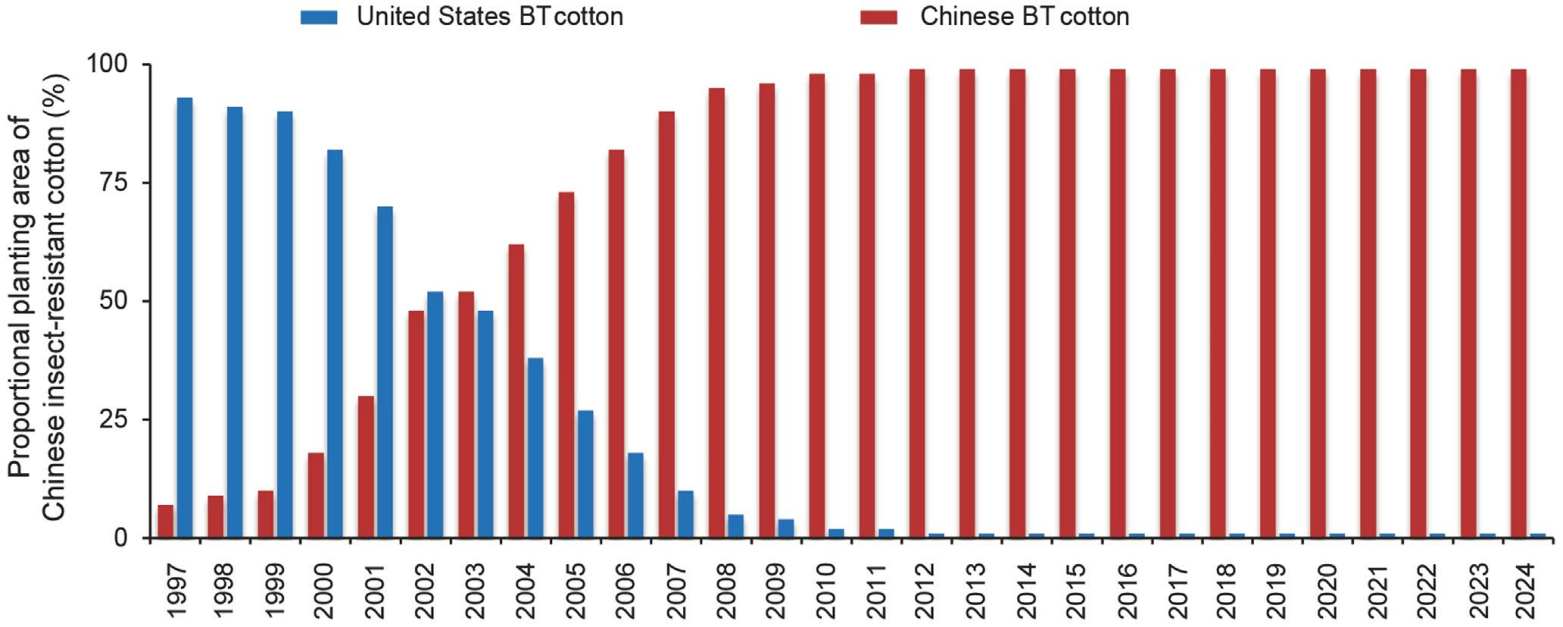
Proportions of Chinese insect‐resistant cotton and United States insect‐resistant cotton planted in China over time. Data were obtained from the National Bureau of Statistics of China (http://www.stats.gov.cn/).

Chinese Bt cotton has not only revolutionised domestic agriculture, but has also had a global impact, serving as an inspiration for agricultural practices worldwide. Through the open sharing of Bt cotton germplasm resources, this technology has been rapidly applied in industry. Because of the diverse range of available varieties, their strong adaptability, and affordable prices, the market share of Chinese Bt cotton increased from 10% in 1999 to 50% in 2003. By 2008, the planting rate of Chinese Bt cotton varieties exceeded 95%, and at present it has surpassed 99%, establishing absolute dominance in the domestic market (Figure 7) (Center et al. 2016; Guo et al. 2015; Sun et al. 2016). As technologies associated with the development of Chinese Bt cotton have matured, the exceptional insect resistance and other outstanding traits of Chinese cotton have garnered attention from cotton‐producing countries worldwide. Collaborative projects for Bt cotton development have been established between China and countries such as India, Australia, and Pakistan (Center et al. 2016). Thus, the success of Chinese Bt cotton has transcended national borders.

The development of Bt cotton in China began in 1997. Compared with other major cotton‐producing countries, China has taken a unique path of “national guidance and independent innovation”. Through strict approval and strategic promotion, and relying on public research institutions, the country has achieved the transformation of technology from introduction to complete autonomy. This model not only ensures the safety of seed use, supports the world's most complete textile industry chain, but also achieves global leadership in total output and high levels of yield per unit area, becoming a successful example of the transformation of agricultural technology under national leadership (Table 1). The research and commercialization of Chinese Bt cotton have paved the way for independent innovation in China's GM cotton industry. This has not only propelled the development of China's cotton industry and related industries but has also challenged the monopoly held by the United States, enhancing China's international competitiveness. The rapid promotion and application of this technology, coupled with its far‐reaching influence, have resulted in significant economic, social, and ecological benefits (Li et al. 2020).

**TABLE 1:** Development comparison of Bt cotton in different countries.

Dimension	Policy and regulation	Technology	Promotion entities	Commercialization start year	Area percentage in 2016	Results and global position	References
China	National strategy‐driven, science‐based regulation supports research and development	From the introduction to self‐innovation	Public research institutes and the government	1997	95.2%	Highest yield per unit area and total production globally	Guo et al. (2015), Zhang and Dong (2024)
India	Initially lagging regulation, later passively standardised	Early reliance on introduction, later diverse local varieties	Private seed companies and farmer autonomous selection	2002	96%	The planting area ranks first globally, but yield per unit area was low	Kumar et al. (2025), Srinivas (2017)
United States	Relatively mature conventional business environment	Original technology leadership, continuous iteration	Multinational agricultural corporations	1996	90.2%	Maintains technological leadership with high and stable yield per unit area	Guo et al. (2015), Zhang and Liu (2025)
Brazil	Initially constrained by legal disputes, later clear regulations promoted development	Adaptive integrated innovation	Large farms and domestic and foreign agricultural enterprises	2005	69.6%	Transformed from a net cotton importer to the world's largest cotton exporter	Bueno et al. (2025), Raphael (2019), Razzaq et al. (2023)

*Note:* The source of the data of Area percentage in 2016 is https://www.isaaa.org/resources/publications/briefs/52/default.asp; the description of Results & Global position is based on: https://apps.fas.usda.gov/psdonline/app/index.html#/app/advQuery.

The production of Chinese Bt cotton is therefore a pivotal milestone in the history of modern agricultural scientific development in China (Pray et al. 2002).

### 5.6 Technological Spillover Effects

The advances in biotechnology associated with Chinese Bt cotton have had additional significant positive effects on other agricultural sectors. First, Bt cotton indirectly controls populations of other pest species by influencing community composition (Lu et al. 2012; Torres et al. 2010; Tabashnik et al. 2013). Reductions in agricultural pest populations promote strong pest management in future seasons. Second, over the past decade, the effectiveness of insect control has improved more than tenfold in cotton and other conventional crops that are extensively cultivated in northern China (including corn, soybean, wheat, and peanut) (Lu et al. 2013; Wu et al. 2008). Third, in transgenic cotton fields, reduced cotton bollworm damage improves plant growth and decreases the necessity for chemical pesticide application (Wu and Guo 2005). This provides favorable nutritional and living conditions for the associated biological community, protecting the cotton field ecosystem (Carrière et al. 2015; Lü et al. 2018; Wu et al. 2002, 2008). The promotion and application of Bt cotton in China not only alleviates the negative impacts of pests on cotton production, but also reduces environmental pollution caused by pesticides, increases farmer income, and plays a crucial role in stabilizing cotton production, ensuring a steady cotton supply and promoting sustainable development of the cotton textile industry in China (Huang, Rozalle, et al. 2002).

The promotion of domestic Bt cotton has also led to positive spillover effects on cotton yield. In recent years, despite stable cotton production in China, the cotton planting area has decreased by~1 500 000 ha (Figure 5) (Mao et al. 2019). This reduction in the cotton planting area has allowed land resources to be used for the cultivation of other food crops, ensuring food security in China. Moreover, the success of Chinese Bt cotton has provided valuable inspiration for the development of crop breeding biotechnologies (Huang et al. 2012). Over the past 30 years, with continuous support via government funding, China has experienced rapid development in both basic and applied research in the field of plant breeding. As a result, the agricultural biotechnology industry has flourished. Importantly, the Chinese Bt cotton effort also nurtured a highly skilled research and development team for crop biotechnology breeding, providing essential talent for the sustainable growth of agricultural breeding in China (Pray et al. 2002; Huang et al. 2012).

## 6 Three Inspirations From Chinese Bt Cotton

The successful development of Chinese Bt cotton has ushered in a prosperous decade for the Chinese cotton industry. However, recent years have brought new challenges to the textile industry because of socioeconomic development and changes in the global landscape. These challenges can be categorised into four key areas. First, rising labor costs warrant an overall improvement in the mechanisation of cotton production and management. Second, increased demands for medium‐ and high‐grade textiles require accelerated cultivation of long‐staple, high‐quality cotton. Third, there is a growing conflict between the needs for grain and for cotton cultivation, necessitating expedited application of biotechnological breeding technologies in cotton production. Finally, to enhance cotton value, it is crucial to explore its untapped potential in various novel applications. To ensure the sustainable development of China's cotton industry and textile sector, it is imperative to prioritise research and advances in the three areas detailed below.

### 6.1 Promoting the Development and Commercialization of Novel Cotton Germplasm

It is imperative to expedite both rigorous safety assessments and the widespread adoption of biotechnologically produced cotton, especially herbicide‐resistant cotton, in agricultural production. Cotton has emerged as a prime example of successful biotechnological breeding (Li et al. 2014). Over the past three decades, the cultivation of Chinese Bt cotton has not only yielded substantial socioeconomic benefits and addressed various challenges in the cotton industry, but has also garnered extensive public support for GM crops in China. At present, weed‐control expenses account for ~25% of labor costs in cotton production (Wang et al. 2015; Zhu et al. 2021). Furthermore, weeds compete with crops for vital resources such as water and nutrients; coupled with fibre contamination during mechanised harvesting, this competition leads to direct economic losses ranging from 10%–20% of the total crop value (Gaur 2014; Holm et al. 1977). Vast coastal saline‐alkali wastelands, such as Yancheng in Jiangsu, Cangzhou in Hebei, and Dongying in Shandong, present new opportunities for expanding the cotton cultivation area. However, cotton cultivation in saline‐alkali lands faces significant challenges, including the eradication of persistent weeds and the development of highly resistant varieties. In recent years, Chinese scientists have made notable progress in developing a series of highly resistant cotton germplasm, including varieties that are resistant to herbicides (Jiang and Wen 2021; Liang, Sun, et al. 2017), *Verticillium* wilt (Wang, Liang, et al. 2020; Wang et al. 2016), drought (Liang, Liu, et al. 2017; Meshram et al. 2022), and saline‐alkali conditions (Abdelraheem et al. 2019; Liang et al. 2016; Meshram et al. 2022). Notably, the herbicide‐resistant cotton variety GGK2 has demonstrated remarkable resistance to glyphosate herbicides with minimal residues (Liang, Sun, et al. 2017). This breakthrough will significantly enhance mechanised weed control in cotton fields and can therefore add substantial economic value.

### 6.2 Accelerating the Application of Modern Biotechnologies in Cotton Genetic Breeding

Advances in cutting‐edge technologies have driven agricultural development into a new era of technological revolutions. In recent years, new methods have significantly improved the efficiency of gene cloning, enabling precisely targeted breeding (Xu et al. 2022). Gene editing techniques have revolutionised crop breeding by allowing customization of desired traits (Gao 2014; Li et al. 2022). Additionally, whole‐genome selection has greatly enhanced breeding efficiency (Desta and Ortiz 2014; Leng et al. 2017). The integration of biotechnologies such as gene editing, transgenic, and whole‐genome selection with modern information technologies such as artificial intelligence has resulted in an efficient agricultural biotechnology system (Wallace et al. 2018; Xu et al. 2022). Such integrative efforts are rapidly transforming and advancing modern breeding technologies, exerting a profound impact on the global biotechnology landscape and the supply of agricultural products.

It is imperative to employ modern biotechnological methods to develop new varieties of cotton that possess desirable traits such as long staple length, superior quality, high yield, resistance to various biotic and abiotic challenges, and suitability for mechanised harvesting. In recent years, continuous enhancements in living conditions both domestically and internationally have caused an unprecedented surge in the demand for medium‐ to high‐end cotton products (OECD/FAO 2021). To bolster the global competitiveness of China's textile industry, it is crucial to fully comprehend fluctuations in the supply and demand dynamics of the cotton textile market and to cultivate long‐staple, high‐quality, and high‐yield cotton. Importation of premium long‐staple cotton to China is severely restricted at present, to a mere few hundred thousand tons per year. Furthermore, out of the whole country, only a small area of Xinjiang is currently suitable for cultivation of long‐staple, high‐quality, high‐yield cotton (Kong et al. 2018). There are significant challenges to expanding the cultivation area. Recently, Chinese scientists harnessed the exceptional fibre quality of *Gossypium barbadense* and the high yield of *Gossypium hirsutum* via hybridization and whole‐genome molecular breeding techniques. Their efforts resulted in the successful development of cotton up to the “double 33” standard, meaning that the fibres are over 33 mm in length and have a tensile strength exceeding 33 eN/tex. This super‐quality cotton variety has the potential to overcome the regional limitations associated with previously released cotton varieties, alleviating the scarcity of long‐staple, high‐quality cotton.

### 6.3 Strengthening Fundamental Cotton Research and Expanding Its Potential Applications

It is crucial to promote application‐oriented fundamental research into the formation and development of vital agronomic traits in cotton. Recent years have seen remarkable progress in fundamental research in crops such as rice, with basic research ultimately leading to breakthroughs in applied studies (Wing et al. 2018; Yu and Li 2021). In contrast, applied research has remained ahead of fundamental research in cotton. The assembly and publishing of complete cotton genome sequences present new opportunities for applied fundamental cotton research (Abid et al. 2022; Yang et al. 2020; Zaidi et al. 2018). However, because of the lack of adequate theoretical research, several pivotal technologies in the cotton industry have remained insufficiently implemented over the past 50 years. For instance, the three‐line hybrid breeding technology not only significantly enhances fibre yield and quality while substantially reducing seed production costs, but also effectively addresses low yield and poor quality in highly insect‐resistant lines via hybrid vigour, achieving synchronised improvements in yield, quality, and resistance (Abid et al. 2022). Nevertheless, understanding of the molecular mechanisms underlying the three‐line hybrid breeding system has significantly lagged behind use of this method, severely impeding large‐scale application of three‐line hybrid cotton. It is therefore imperative to strengthen fundamental research into crucial traits such as three‐line hybrid fertility, fibre development, drought resistance, and salt tolerance to reinforce theoretical innovation and technological reserves. This will be vital in ensuring long‐term maintenance of advantages in China's cotton industry.

It is crucial to explore the untapped potential of cotton and expand its applications through open collaboration and knowledge sharing. Cotton is a versatile resource; cotton fibre is the most consumed natural fibre in the world and cottonseed oil is the fifth most abundantly produced edible vegetable oil worldwide (Bellaloui et al. 2015; Mehboob ur et al. 2012). Short cotton fibres also serve as a vital raw material for industries such as national defence, textiles, chemical fibres, and papermaking. Moreover, cottonseed meal contains ~44% crude protein, ~1% phosphorus, and rich reserves of vitamin E, thiamine, and B vitamins. These characteristics make it a high‐calorie, biologically valuable ingredient for protein feed. Cotton also contains gossypol, a naturally occurring toxic substance that poses a challenge to the use of cottonseed oil and cottonseed meal as animal feed (Lin et al. 2023). Gossypol primarily acts as a defence compound, protecting cotton plants against diseases and pests; some research has even suggested potential effects of this compound in cancer treatments (Wang, Li, Wen, et al. 2022). Biotechnologies could be applied to enhance the gossypol content of cotton leaves and stems, bolstering plant resistance to diseases and pests while simultaneously preventing gossypol synthesis in the seeds. This would enable cottonseeds to be consumed as a food source, similar to chickpeas, and utilised as feed for poultry and livestock.

In summary, the Chinese Bt cotton project (Pray et al. 2002), which was launched three decades ago, continues to offer valuable lessons for China's biological breeding efforts. Chinese Bt cotton has proven to be highly successful, boasting an impressive track record of safety, efficacy, and environmental benefits. Additionally, it has played a crucial role in driving the prosperity of the cotton and textile industries in China. The story of Chinese Bt cotton serves as a valuable reminder of the importance of dedication, an interdisciplinary approach, and consistent funding for advances in biotechnological crop breeding methods (Huang, Rozalle, et al. 2002; Pray et al. 2002). Future crop breeding efforts will be well served by these lessons, allowing continued technological advances that benefit farmers, consumers, ecosystems, and the country as a whole.

## 7 Conclusions and Future Prospects

Insect–plant interactions have co‐evolved for over 400 million years (Labandeira 2013) and remain a major challenge in crop protection. Research on these interactions mainly focuses on plant defence mechanisms, plant–insect communication, and insect counter‐defence strategies (Wang, Xu, Yang, Liu, et al. 2024; Benelli and Maggi 2022; Lai et al. 2022). Bt cotton represents one of the most successful applications in this field, substantially reducing losses caused by bollworms, although secondary pests such as aphids, thrips, mirids, and whiteflies have subsequently emerged (Zhao et al. 2011; Zeilinger et al. 2016). Meanwhile, the rapid development of genomics and other omics technologies since the Human Genome Project has provided new tools and opportunities for advancing pest management research.

Through genome assembly and data mining, key genes associated with feeding preference, environmental adaptation, signal transduction, growth, and development have been identified in various pest species (Ashraf et al. 2018; Li, Zhao, et al. 2019). These genes can be broadly categorised as direct‐acting and indirect‐acting. Direct‐acting genes directly influence herbivorous feeding behaviour (e.g., detoxification and chemosensation), whereas indirect‐acting genes affect traits such as stress tolerance, diapause, body shape diversity, and reproductive mode that in turn modify feeding potential. For example, Jin, North, et al. (2023) showed that a nonsynonymous SNP in Tret1 enhances cold‐induced diapause in *Helicoverpa armigera*, indirectly improving feeding capacity under low temperatures. In aphids, comparative genomics of eight species indicates that parthenogenesis accelerates chromosomal recombination and enhances adaptive evolution, thereby indirectly supporting pest outbreaks (Huang et al. 2025). Compared to indirect action genes, direct action genes have been more extensively studied. Genomic studies have identified genes related to omnivory (Li, Yang, et al. 2017), detoxification resistance (Xu et al. 2023; Xia et al. 2021; Rotenberg et al. 2020), and chemosensation in pests (Quan et al. 2019; Zhang, Gao, et al. 2022), which directly regulate insect feeding on host plants. Overall, genomic analyses have expanded the mechanistic understanding of insect–plant interactions and continue to provide new targets and conceptual frameworks for pest management.

Although insect genome research has achieved considerable success, with chromosome‐level genomes accounting for one‐third of the published insect genomes, T2T‐level insect genomes are rarely mentioned (Li et al. 2025; Li, Xiao, et al. 2024). Therefore, high‐quality insect genome assembly remains a challenge to be solved. Insects are small, which often necessitates pooling individuals to obtain sufficient DNA, resulting in elevated heterozygosity. Contaminants from symbiotic bacteria and viruses further compromise DNA quality and assembly accuracy. Consequently, insect genome assemblies are generally less contiguous than those of plants or vertebrates. Moreover, limited functional annotation resources and incomplete reference databases hinder standardised genome annotation, which explains why many published insect genomes still lack accompanying mitochondrial, rDNA, or complete gene annotation information (Li, Xiao, et al. 2024; Wang and Wang 2023). Recently, Pacific Biosciences (PacBio) has launched a new technology, Ampli‐Fi ultra‐low‐input protocol, which can perform high‐quality HiFi sequencing with as little as 1 ng of DNA (Bein et al. 2025; Männer et al. 2024). This may solve the problem of DNA quality when sampling pests. Moreover, with the development of artificial intelligence (AI) algorithms, it has become possible to simplify the relatively cumbersome gene annotation process. We look forward to the joint progress of various types of omics technologies and their corresponding bioinformatics algorithms to solve the problem of higher‐quality insect genome assembly and meet the increasingly in‐depth needs of pest control research.

In fact, the methods for cotton insect resistance research in the post‐Bt era are diverse. Initially, the discovery of Bt insect‐resistant proteins required a lot of experimental costs and time. This situation may change in the near future with the development of various technologies. On the basis of high‐quality DNA sequencing data and experimental data on the physicochemical properties of proteins, the use of AI for the design and creation of cheaper and faster insecticidal proteins and small peptides, as well as for the precise prediction of insect effectors and their targets, has gradually become feasible with the support of new‐generation algorithms (Notin et al. 2024). In addition, integrating artificial intelligence into the directional and assisted evolution of microorganisms to screen for new insect‐resistant proteins will also be an important direction in the future (Wang, Xue, et al. 2021; Zhang et al. 2024). Finally, guided by synthetic biology, combined with high‐quality genomic data to screen for endogenous or exogenous key genes, choosing different types of gene editing tools to achieve more accurate and efficient targeted gene editing, and artificially synthesising or modifying gene pathways to endow crops with new biological characteristics will inject new vitality into the creation of new Bt cotton (de Lange et al. 2018; Liu and Stewart 2016; Schiemann et al. 2019). In summary, we will employ various feasible technologies and methods to achieve more intelligent, rapid, and precise cotton insect‐resistant breeding.

## Author Contributions

All authors contributed to the preparation of the manuscript. C.L. and S.G. conceptualised the review outline; C.L., Z.M., X.H., and G.Y. wrote the original draft; C.L., X.H., and G.Y. prepared the figures; C.L., S.J., X.H., Y.W., Y.Z., Q.Z., and Y.L. provided constructive feedback for the original draft and figures; C.L., S.J., Z.M., and X.H. reviewed and edited the manuscript.

## Funding

This work is supported by the Biological Breeding‐National Science and Technology Major Project (2023ZD04039‐03‐3, 2023ZD04076, NK202201020302), National Key Research and Development Program of China (2023YFF1000103), the Agricultural Gene Editing Platform Technology and Breeding R&D, Hubei (2024BBA001), Xinjiang Tianshan Innovation Team (2025D14001), Xinjiang Tianchi Elite Distinguished Expert to C.L., Agricultural Science and Technology Innovation Program of CAAS to C.L., and Basic Research Funding of BRI, CAAS to C.L.

## Conflicts of Interest

The authors declare no conflicts of interest.

## Supporting information


**Table S1:** Bt pesticidal protein type and the orders in the taxonomic classification of their target pests.

## Data Availability

Data sharing is not applicable to this article as no datasets were generated or analysed during the current study.
