# Supplementary material for: Three Decades of China's Bt Cotton: Achievements and Insights
Source: Plant Biotechnol J. 2026 Mar 18;24(6):4393–414. doi: 10.1111/pbi.70641 (PMC13205746; doi:10.1111/pbi.70641)
Supplement: Supplementary file 1 — Table S1: Bt pesticidal protein type and the orders in the taxonomic classification of their target pests. [file PBI-24-4393-s001.docx]

**Table S1** Bt pesticidal protein type and the orders in the taxonomic classification of their target pests

| **Structural**  **classes** | ***Bacillus thuringiensis***  **pesticidal protein** | **Orders of**  **target pests** | **References** |
| --- | --- | --- | --- |
| Cry | Cry1A-K,Cry2A,Cry3,cry3Bb1,Cry7B,Cry8D  Cry9A-C,E,Cry22A,Cry32A  Cry1A-F,I,J,Cry3A-C,Cry7A  Cry8A-G,K,N,S,Cry9B,D,Cry10A,Cry14A  Cry22A-B,Cry43A-B  Cry1Ab,Cry1Ac,Cry1Ba,Cry1Cb2,Cry2Aa  Cry3Aa,Cry4Aa,Cry11A,Cry73Ba,Cry5Ba2  Cry7Ca1  Cry1A-C,Cry2A,Cry4A-B,Cry10A,Cry11A-B  Cry19A-B,Cry20A,Cry24B-C,Cry27A,Cry30C  Cry32B,Cry39A,Cry44A,Cry47A,Cry48A,Cry59A  Cry5A-B,Cry12A,Cry13A,Cry14A,Cry21A  Cry31Aa,Cry73Aa | Lepidoptera  Coleoptera  Hemiptera  Orthoptera  Diptera  Rhabditida | Palma *et al*. (2014a), Zafar *et al*. (2022)  Palma *et al*. (2014a)  Domínguez-Arrizabalaga *et al*. (2020), Palma *et al*. (2014a)  Walters and English, (1995)  Porcar *et al*. (2009), Paula and Andow (2016), Palma *et al*. (2014a), Palma *et al*. (2014b), Torres-Quintero *et al*. (2022), Zhao *et al*. (2020), Wang *et al*. (2024)  Wu *et al*., (2011)  Valtierra-de-Luis *et al*. (2020)  Noguera and Ibarra (2010)  Palma *et al*. (2014a)  Wei *et al*. (2003)  Liang *et al*. (2022) |
| Cyt | Cyt1A,Cyt2C  Cyt1Aa  Cyt1A-B,Cyt2A-B | Coleopteran Hemiptera  Diptera | van Frankenhuyzen, (2009)  Porcar *et al*. (2009)  Soberón *et al.* (2013) |
| Vip3 | Vip3A,Vip3Aa,Vip3Ca  Vip3 | Lepidoptera  Hemiptera | Lone *et al*. (2016), Yang *et al*. (2018)  Syed *et al*. (2020)  El-Gaied *et al*. (2020) |
| Vpb/Vpa | Vip1Aa/Vip2Aa,Vip1Aa/Vip2Ab Vip1Ad/Vip2Ag,Vip1Ba/Vip2Ba  Vip1Bb/Vip2Bb,Vip1Da/Vip2Ad  Vip1Ac1/Vip2Ae3  Vip1Ae/Vip2Ae | Coleoptera  Hemiptera | Domínguez-Arrizabalaga *et al*. (2020)  Yu *et al*. (2011)  Sattar and Maiti (2011) |
| App | App6A-B | Rhabditida | Yu *et al*. (2014) |
| Mpp | Mpp15Aa  Mpp1Aa(Sip1Aa),Mpp1Ab(Sip1Ab),Mpp51Aa  Mpp51Aa,Mpp64Ba/Ca  Mpp51Aa  Mpp60 | Lepidoptera  Coleoptera  Hemiptera  Thysanoptera  Diptera | Palma *et al*. (2014a)  Donovan *et al*. (2006),  Bachman *et al*. (2017)  Graham *et al,* (2018)  Bachman *et al*. (2017)  Sun *et al*. (2013) |
| Gpp/Tpp | Gpp34/Tpp35 | Coleoptera | Baum et al., 2004) |
| Tpp | Tpp36A  Tpp78Aa1,Tpp78Ab1,Tpp78Ba1,Tpp78Bb1  Tpp78Ca1,Tpp78Da1, Tpp78Ae1,Tpp80Aa3  Tpp80Ac1,Tpp80Ad1  Tpp49 | Coleoptera  Hemiptera  Diptera | Palma *et al*. (2014a)  Wang *et al*. (2018)  Cao *et al*. (2020)  Cao *et al*., 2023)  Palma *et al*. (2014a) |
| Xpp | Xpp37Aa(Mpp23Aa/Xpp37Aa),Xpp55Aa | Coleoptera | Domínguez-Arrizabalaga *et al*. (2020) |

**References**

Bachman, P.M., Ahmad, A., Ahrens, J.E., Akbar, W., Baum, J.A., Brown, S., Clark, T.L.,*et al.* (2017) Characterization of the Activity Spectrum of MON 88702 and the Plant-Incorporated Protectant Cry51Aa2.834_16. *PLoS One* **12**, e0169409.

Baum, J.A., Chu, C.R., Rupar, M., Brown, G.R., Donovan, W.P., Huesing, J.E., Ilagan, O., *et al.* (2004) Binary toxins from *Bacillus thuringiensis* active against the western corn rootworm, Diabrotica virgifera virgifera LeConte. *Appl. Environ. Microbiol.* **70**, 4889-4898.

Cao, B., Shu, C., Geng, L., Song, F. and Zhang, J. (2020) Cry78Ba1, One Novel Crystal Protein from *Bacillus thuringiensis* with High Insecticidal Activity against Rice Planthopper. *J. Agric. Food Chem.* **68**, 2539-2546.

Cao, B., Sun, X., Shu, C., Geng, L. and Zhang, J. (2023) Identification and functional characterization of eight novel tpp family genes from *Bacillus thuringiensis*. *Pest Manag. Sci.* **79**, 4244-4253.

Domínguez-Arrizabalaga, M., Villanueva, M., Escriche, B., Ancín-Azpilicueta, C. and Caballero, P. (2020) Insecticidal Activity of Bacillus thuringiensis Proteins Against Coleopteran Pests. *Toxins (Basel)* **12**, 430.

Donovan, W.P., Engleman, J.T., Donovan, J.C., Baum, J.A., Bunkers, G.J., Chi, D.J., Clinton, W.P., *et al*. (2006) Discovery and characterization of Sip1A: A novel secreted protein from *Bacillus thuringiensis* with activity against coleopteran larvae. *Appl. Microbiol. Biotechnol.* **72**, 713-719.

El-Gaied, L., Mahmoud, A., Salem, R., Elmenofy, W., Saleh, I., Abulreesh, H.H., Arif, I.A. *et al*. (2020) Characterization, cloning, expression and bioassay of *vip3* gene isolated from an Egyptian *Bacillus thuringiensis* against whiteflies. *Saudi J. Biol. Sci.* **27**, 1363-1367.

Erban, T., Nesvorna, M., Erbanova, M. and Hubert, J. (2009) *Bacillus thuringiensis* var. tenebrionis control of synanthropic mites (Acari: Acaridida) under laboratory conditions. *Exp. Appl. Acarol.* **49**, 339-346.

Graham, S. H., and Stewart, S. D. (2018) Field Study Investigating Cry51Aa2.834_16 in Cotton for Control of Thrips (Thysanoptera: Thripidae) and Tarnished Plant Bugs (Hemiptera: Miridae). *J. Econ. Entomol.* **111**, 2717–2726.

Liang, Z., Ali, Q., Wang, Y., Mu, G., Kan, X., Ren, Y., Manghwar, H. *et al*. (2022) Toxicity of *Bacillus thuringiensis* Strains Derived from the Novel Crystal Protein Cry31Aa with High Nematicidal Activity against Rice Parasitic Nematode *Aphelenchoides besseyi*. *Int. J. Mol. Sci.* **23**, 8189.

Lone, S.A., Yadav, R., Malik, A. and Padaria, J.C. (2016) Molecular and insecticidal characterization of Vip3A protein producing *Bacillus thuringiensis* strains toxic against *Helicoverpa armigera* (Lepidoptera: Noctuidae). *Can. J. Microbiol.* **62**, 179-190.

Noguera, P.A. and Ibarra, J.E. (2010) Detection of new cry genes of *Bacillus thuringiensis* by use of a novel PCR primer system. *Appl. Environ. Microbiol.* **76**, 6150-6155.

Palma, L., Muñoz, D., Berry, C., Murillo, J. and Caballero, P. (2014a) *Bacillus thuringiensis* toxins: an overview of their biocidal activity. *Toxins (Basel)* **6**, 3296-3325.

Palma, L., Muñoz, D., Berry, C., Murillo, J., de Escudero, I.R. and Caballero, P. (2014b) Molecular and insecticidal characterization of a novel Cry-related protein from *Bacillus thuringiensis* toxic against *Myzus persicae.* *Toxins (Basel)* **6**, 3144-3156.

Paula, D.P. and Andow, D.A. (2016) Differential Cry toxin detection and effect on *Brevicoryne brassicae* and *Myzus persicae* feeding on artificial diet. ***Entomol. Exp. Appl.*** **159**, 54-60.

Porcar, M., Grenier, A.M., Federici, B. and Rahbé, Y. (2009) Effects of *Bacillus thuringiensis* delta-endotoxins on the pea aphid (*Acyrthosiphon pisum*). *Appl. Environ. Microbiol.* **75**, 4897-4900.

Sattar, S. and Maiti, M.K. (2011) Molecular characterization of a novel vegetative insecticidal protein from *Bacillus thuringiensis* effective against sap-sucking insect pest. *J. Microbiol. Biotechnol.* **21**, 937-946.

Soberón, M., López-Díaz, J.A. and Bravo, A. (2013) Cyt toxins produced by *Bacillus thuringiensis*: a protein fold conserved in several pathogenic microorganisms. *Peptides* **41**, 87-93.

Sun, Y., Zhao, Q., Xia, L., Ding, X., Hu, Q., Federici, B.A. and Park, H.W. (2013) Identification and characterization of three previously undescribed crystal proteins from *Bacillus thuringiensis* *subsp. jegathesan*. *Appl. Environ. Microbiol.* **79**, 3364-3370.

Syed, T., Askari, M., Meng, Z., Li, Y., Abid, M.A., Wei, Y., Guo, S. *et al*. (2020) Current Insights on Vegetative Insecticidal Proteins (Vip) as Next Generation Pest Killers. *Toxins (Basel)* **12**, 522.

Torres-Quintero, M.C., Arenas-Sosa, I., Zuñiga-Navarrete, F., Hernández-Velázquez, V.M., Alvear-Garcia, A. and Peña-Chora, G. (2022) Characterization of insecticidal Cry1Cb2 protein from *Bacillus thuringiensis* toxic to Myzus persicae (Sulzer). *J. Invertebr. Pathol.* **189**, 107731.

Valtierra-de-Luis, D., Villanueva, M., Lai, L., Williams, T. and Caballero, P. (2020) Potential of Cry10Aa and Cyt2Ba, Two Minority δ-endotoxins Produced by *Bacillus thuringiensis* ser*. israelensis*, for the Control of *Aedes aegypti Larvae*. *Toxins (Basel)* **12**, 355.

van Frankenhuyzen, K. (2009) Insecticidal activity of *Bacillus thuringiensis* crystal proteins. *J. Invertebr. Pathol.* **101**, 1-16.

Walters, F.S. and English, L.H. (1995) Toxicity of *Bacillus thuringiensis* δ- endotoxins toward the potato aphid in an artificial diet bioassay. *Entomol. Exp. Appl. et Applicata* **77**, 211-216.

Wang, Y., Liu, Y., Zhang, J., Crickmore, N., Song, F., Gao, J. and Shu, C. (2018) Cry78Aa, a novel *Bacillus thuringiensis* insecticidal protein with activity against *Laodelphax striatellus* and *Nilaparvata lugens*. *J. Invertebr. Pathol.* **158**, 1-5.

Wang, Y., Wang, M., Zhang, Y., Chen, F., Sun, M., Li, S., Zhang, J. *et al*. (2024) Resistance to both aphids and nematodes in tobacco plants expressing a *Bacillus thuringiensis* crystal protein. *Pest Manag Sci* **80**, 3098-3106.

Wei, J.Z., Hale, K., Carta, L., Platzer, E., Wong, C., Fang, S.C. and Aroian, R.V. (2003) *Bacillus thuringiensis* crystal proteins that target nematodes. *Proc. Natl. Acad. Sci. U S A* **100**, 2760-2765.

Wu, Y., Lei, C., Yi, D., Liu, P. and Gao, M. (2011) Novel *Bacillus thuringiensis* δ-endotoxin active against *Locusta migratoria manilensis*. *Appl. Environ. Microbiol.* **77**, 3227-3233.

Yang, J., Quan, Y., Sivaprasath, P., Shabbir, M.Z., Wang, Z., Ferré, J. and He, K. (2018) Insecticidal Activity and Synergistic Combinations of Ten Different Bt Toxins against *Mythimna separata* (Walker). *Toxins (Basel)* **10**, 454.

Yu, X., Liu, T., Liang, X., Tang, C., Zhu, J., Wang, S., Li, S. *et al*. (2011) Rapid detection of *vip1*-type genes from *Bacillus cereus* and characterization of a novel *vip* binary toxin gene. *FEMS Microbiol. Lett.* **325**, 30-36.

Yu, Z., Luo, H., Xiong, J., Zhou, Q., Xia, L., Sun, M., Li, L. *et al*. (2014) *Bacillus thuringiensis* Cry6A exhibits nematicidal activity to Caenorhabditis elegans bre mutants and synergistic activity with Cry5B to C. elegans. *Lett. Appl. Microbiol.* **58**, 511-519.

Zafar, M.M., Mustafa, G., Shoukat, F., Idrees, A., Ali, A., Sharif, F., Shakeel, A. *et al*. (2022) Heterologous expression of *cry3Bb1* and *cry3* genes for enhanced resistance against insect pests in cotton. *Sci. Rep.* **12**, 10878.

Zhao, X., Zhang, B., Fu, L., Li, Q., Lin, Y. and Yu, X. (2020) Possible Insecticidal Mechanism of Cry41-Related Toxin against *Myzus persicae* by Enhancing Cathepsin B Activity. *J. Agric. Food. Chem.* **68**, 4607-4615.
